# Sleep quality and duration following the use and co-use of alcohol and cannabis in the daily life of community adults

**DOI:** 10.1016/j.drugalcdep.2025.112946

**Published:** 2025-10-25

**Authors:** Melissa Nance, Mary Beth Miller, Jarrod M. Ellingson, Ryan W. Carpenter

**Affiliations:** a University of Notre Dame, Department of Psychology, Notre Dame, IN, USA; b University of Missouri, Department of Psychiatry, 1 Hospital Drive DC067.00, Columbia, MO 65212, USA; c University of Colorado School of Medicine, Department of Psychiatry, Aurora, CO, USA

**Keywords:** Sleep quality, Sleep duration, Alcohol use, Cannabis use, Concurrent use, Co-use

## Abstract

**Background:**

Sleep-promotion is one of the most common reasons people use cannabis. Previous studies, primarily among young adults, suggest that cannabis use has negligible day-level effects on sleep but may attenuate alcohol’s negative effects when both substances are used. Studies among middle-aged/older adults are needed to determine the generalizability of these day-level associations.

**Methods:**

This preregistered secondary data analysis examined daily-life associations between alcohol and cannabis use with next-day sleep quality and duration in a community sample of 48 adults (50 % female; age *M*=36.42, *SD*=10.96) reporting weekly alcohol and/or cannabis use. Participants completed ecological momentary assessments over 60 days. Multilevel models examined whether alcohol use, cannabis use, or alcohol-cannabis co-use (vs. no use) that day were associated with sleep quality and duration that night. Next, sleep quality and duration were examined as predictors of next-day craving and alcohol/cannabis use.

**Results:**

Cannabis use was associated with longer sleep duration than days of alcohol use, co-use, or no use, which were not different from one another. Sleep quality did not differ following alcohol/cannabis use. Sleep quality and duration were not associated with next-day craving for alcohol or cannabis. Longer sleep duration was associated with higher likelihood of next-day cannabis use.

**Conclusions:**

Community adults report sleeping ~15 min longer on days of cannabis use, unless they also drank alcohol that day. This finding underscores the importance of examining co-use in daily life. Cannabis use demonstrated limited benefits for sleep, which should be evaluated in context with risk for increasing cannabis use over time.

## 1. Introduction

Rates of cannabis use continue to rise in the U.S., as more states legalize medical and non-medical cannabis use (Caulkins, 2024). Sleep-promotion is one of the most common reasons that individuals report for using cannabis, regardless of whether or not they receive cannabis through a medical program (Berey et al., 2023; Bohnert et al., 2018; Walsh et al., 2013). However, much remains unknown about how cannabis use influences sleep. There are pharmacological reasons to believe that cannabis may help with sleep, as plant-based cannabinoids, such as delta-9 tetrahydrocannabinol (THC) and cannabidiol (CBD), bond with the two main endocannabinoid receptors (CB1 and CB2). These receptors are located throughout the brain and peripheral nervous system and are linked to the regulation of circadian rhythm, in part by increasing adenosine levels, a sleep-promoting agent (Babson et al., 2017; Babson and Bonn-Miller, 2014; Murillo-Rodriguez et al., 2003). As plant-based cannabinoids (e.g., THC and CBD) act on the same receptors as endogenous cannabinoids, they could also induce sleepiness, supporting the widely-held belief that cannabis will help with falling and staying asleep (Berey et al., 2023).

Some research suggests cannabis may, at least in the short term, reduce sleep onset latency (Sznitman et al., 2020) and increase sleep quality among adults initiating use for medical purposes (Tervo-Clemmens et al., 2022) and those with clinical symptoms (Davis et al., 2025). People with sleep problems who use cannabis tend to report that cannabis helps them fall asleep faster and stay asleep longer; however, subjective sleep improvements often occur in the context of increased frequency of cannabis use, raising the risk for cannabis use disorder (Altman et al., 2019; Tervo-Clemmens et al., 2022). Moreover, other studies indicate cannabis use is associated with more actigraphy-assessed nighttime awakenings (Bell et al., 2022), less slow-wave (“deep”) sleep (Nicholson et al., 2004), and more next-day fatigue/sleepiness among both clinical and non-clinical samples (Goodhines et al., 2019; Gorelick et al., 2013; Nicholson et al., 2004). Collectively, data suggest that cannabis may improve some dimensions of sleep while simultaneously disrupting others.

In comparison to cannabis use, a larger body of research has examined the impacts of alcohol use on sleep. Across polysomnography and self-report studies, alcohol use has been associated with disrupted sleep architecture and sleep-related respiratory issues resulting in sleep fragmentation, early awakening, and reduced sleep duration (Ebrahim et al., 2013; Kolla et al., 2018). Furthermore, alcohol use has been associated with insomnia and other sleep disorders (Chakravorty et al., 2016). Despite well-established associations between alcohol and disrupted sleep, alcohol is used as a sleep-aid by about 18 % of adults with insomnia (Morin et al., 2024) and 35 % of heavy-drinking adults with insomnia (Miller et al., 2025). Beliefs around the effectiveness of alcohol use as a sleep-aid may be developed because low doses of alcohol (<3 standard drinks) prior to bed may initially reduce sleep onset latency, but these effects appear to diminish after 3–6 days of continued alcohol use (Roehrs 2018; Stein 2006; Stein and Friedmann, 2006).

Given the common use and perceived effectiveness of both substances as sleep aids, there is a need to understand whether same-day use of both substances (i.e., alcohol-cannabis co-use) impacts sleep that night. There may be synergistic effects (e.g., even stronger effects on sleep onset latency), but it is possible that co-use of alcohol and cannabis affects sleep differently than use of either substance alone. Co-use of cannabis and alcohol is prevalent and associated with additive impairment effects, greater overall consumption, and greater likelihood of mental health and substance use disorders (Yurasek et al., 2017). However, to our knowledge, only three studies have examined day-level associations between alcohol-cannabis co-use and sleep. All three found that alcohol-only use was associated with worsening of at least one sleep parameter (e.g., worse sleep quality, more nighttime awakenings, more next-day impairment; Graupensperger et al., 2021; Sznitman et al., 2023; Wycoff et al., 2024), while results for cannabis differed across studies. Cannabis use was associated with better same-night sleep parameters (e.g., shorter sleep onset, longer sleep duration, fewer awakenings, better sleep quality) among those who used on roughly 30–40 % of study days (Graupensperger et al., 2021; Sznitman et al., 2023) but was not significantly associated with same-night sleep among those who used almost daily (Wycoff et al., 2024). Interestingly, all three studies found that the negative association between alcohol and same-night sleep parameters was weaker on days that alcohol was combined with cannabis (Graupensperger et al., 2021; Sznitman et al., 2023; Wycoff et al., 2024). These studies offer initial evidence that co-use may be associated with less impairment than alcohol use alone, but the generalizability of these findings remains unclear, as two of the three studies examined young adults exclusively. Available evidence suggests that circadian and cannabinoid systems change with age and, in particular, that middle-aged/older adults who use cannabis may show better sleep efficiency and duration than younger adults who use cannabis (Hodges and Ashpole, 2019; Winiger et al., 2021). Thus, there may be important differences in how sleep is influenced by alcohol-cannabis co-use in middle and older adulthood.

It is also possible that sleep quality and duration impact whether people crave and use cannabis and alcohol later that day. Experiencing craving, or a strong urge/desire to use, is part of the diagnostic criteria for substance use disorder and is a proximate measure of risk for engaging in a use episode (American Psychiatric Association, 2013; King et al., 2025; Waddell et al., 2021). Insufficient sleep may influence one’s mood and cognitive resources, increasing the likelihood of experiencing craving and also reducing willpower to resist using alcohol or cannabis (Freeman and Gottfredson, 2018). Thus, it is important to understand the role of sleep on both subsequent craving and use. However, research on this topic is mixed. Young adults have reported greater craving for alcohol and cannabis after nights with shorter sleep duration (Graupensperger et al., 2022). However, other studies have linked more trouble sleeping to lower likelihood of cannabis use (Berey et al., 2024) and better sleep (longer sleep duration) to increased next-day craving and odds of use (Wycoff et al., 2024). Similarly, at least two studies have linked better sleep (quality and efficiency) to heavier next-day drinking (Fucito et al., 2018; Miller et al., 2021).

### 1.1. Current study

Despite initial work examining alcohol-cannabis co-use and sleep in daily life, the exact impact of combining these substances remains unclear, even as rates of co-use continue to increase. Given that alcohol has established negative impacts on sleep, while available evidence suggests both potential positives and negatives of cannabis, there is a need to clarify this relationship. In particular, it is important to know whether cannabis may potentially mask the negative impacts of alcohol (e.g., increasing perceived sleep quality, but not duration). Additionally, previous studies on co-use have focused primarily on young adults, and research in post-college adults is needed. This secondary data analysis fills this gap in the literature by testing real-life associations of alcohol/cannabis use with subsequent sleep quality and sleep duration in a community sample of 48 adults who reported at least weekly alcohol and/or cannabis use in the past month.

### 1.2. Primary hypotheses

First, given previous work (Sznitman et al., 2020; Tervo-Clemmens et al., 2022), we hypothesized that days of cannabis-only use would be related to better next-morning sleep quality and longer self-reported sleep duration than days with alcohol-only use and days with no cannabis/alcohol use. Second, we hypothesized that days of alcohol-only use would be related to lower next-morning sleep quality and shorter sleep duration than days with no alcohol/cannabis use. Finally, we hypothesized that cannabis would moderate day-level associations between alcohol use and sleep parameters, such that alcohol use would be less strongly associated with sleep parameters on days that cannabis was also used.

### 1.3. Secondary hypotheses

How sleep impacts later craving for and use of cannabis and alcohol is unclear based on prior work. We hypothesized that sleep quality and duration would be inversely associated with next-day craving for alcohol use and craving for cannabis use. We additionally hypothesized that lower sleep quality and shorter sleep duration would be associated with greater odds of next-day alcohol and/or cannabis use (vs. no use). Our pre-registration proposed examination of next-day use of either alcohol or cannabis use (in a single model), as both substances have perceived sleep-related benefits and we anticipated that either substance or their co-use would be more likely following nights with worse sleep, potentially due to sleep-related motives for use. Though not preregistered, we also examined next-day alcohol use and cannabis use as separate outcomes.

## 2. Method

Procedures were approved by the Colorado Multiple Institutional Review Board (Protocol #20–1002). Recruitment occurred via online advertisements from July 2020 to July 2021 (during the COVID-19 pandemic). Advertisements were posted on Craigslist for “a research study to learn more about the impact of COVID-19 on mental health and substance use in adults.” Potential participants were informed that they would complete an online baseline survey and that some participants may also be asked to complete brief text-message based surveys every day for 60 days after the baseline survey. Eligibility criteria included being age 21 or older (i.e., of legal age to obtain alcohol and cannabis) and living in the state of Colorado as verified by photo of their driver’s license or other state-issued identification. For the ecological momentary assessment (EMA) portion of the protocol, participants also had to report at least weekly alcohol and/or cannabis use in the past month. Recruitment targeted those reporting regular use of alcohol/cannabis use (a) to ensure sufficient instances of alcohol/cannabis use throughout the EMA protocol and (b) to characterize day-level associations among those who likely have some level of physical tolerance and experience greater risk for alcohol/cannabis-related problems.

Of the 64 adults invited to participate in EMA, 2 declined, 3 agreed but did not follow up, and 11 did not respond, resulting in a sample of 48 adults. We initially invited participants to enroll in the EMA study until we reached 45 enrolled participants (24 females, 21 males) and then recruited 3 additional males to balance on sex. Older and non-White participants were also prioritized to increase the generalizability of findings.

### 2.1. Protocol

Following informed consent, participants were given instructions on how to complete the EMA assessments and could practice answering questions with a research assistant via Zoom to ensure they understood procedures. Participants completed a 60-day EMA protocol involving at least four daily assessments that were delivered within four different time windows: morning (0700–1100), afternoon (1100–1600), evening (1600–1900), and night (1900–2200). Text message notifications included a REDCap survey link. Reminders were sent 30 and 60 min later and the survey closed after two hours. Evening surveys asked, “Do you plan to use marijuana or alcohol later tonight?” When participants reported “Yes,” “Not sure,” or reported alcohol/cannabis use in the nighttime survey, they received additional assessments every two hours. Once an additional survey went two hours without response, no further surveys were sent that night.

Compensation was based on the number of assessments completed and increased over the course of the EMA period. Participants received $25 for completion of the baseline survey. For the duration of the EMA protocol, they earned $1 per survey completed. Bonus payments became available at increasing increments as the study progressed: on days 16–30, participants earned a $1 bonus for each day that they completed 4 + surveys; on days 31–45, they earned a $2 bonus for each day of 4 + surveys; and on days 46–60, they earned a $3 bonus for each day of 4 + surveys. Thus, participants could earn up to $330. Additionally, two raffle drawings were held, one for participants that completed 90 % of surveys over days 1–30 and one for days 31–60. Participants could enter both drawings, and three winners from each drawing received an additional $100.

### 2.2. Baseline measures

The baseline survey included the 10-item Alcohol Use Disorder Identification Test (AUDIT; Saunders et al., 1993), the 8-item Cannabis Use Disorder Identification Test (CUDIT-R; Adamson et al., 2010), and the Pittsburgh Sleep Quality Index (PSQI; Buysse et al., 1989), along with other self-report measures of psychopathology and related constructs that were not included in analyses.

### 2.3. EMA measures

#### 2.3.1. Alcohol/cannabis use

At each prompt, participants reported use as follows: “Since your last response…” (1) “If you used alcohol, about how many standard drinks did you have (i.e., 12 oz 4 % ABV beer, 6 oz of wine, or 1.5 oz of liquor)?” Participants could report from 0 to 9 + drinks. (2) “If you used marijuana, what type(s) of marijuana did you use?” Participants could choose all that applied from: none, flower/dry herb, edibles, concentrates (e.g., dabbing, shatter, wax, etc.), and other. If participants reported cannabis use, they were asked to report on the concentration of THC in “the marijuana flower that you smoked” and/or the “concentrates that you used.” Participants were provided with a range of concentrations (0–4 %, 5–9 %, etc.) with response options tailored to the product type (flower vs. concentrates) along with a “don’t know” option. For flower, the final response was “Greater than 30 %” and for concentrates it was “90 % or more.” In analyses, the midpoint of each THC potency response category was used as the specific potency value. For the upper-bound response categories, values of 32.5 % and 94.5 % were assigned, respectively, for flower and concentrate products. Responses indicating “don’t know” for THC potency were treated as missing and excluded from the main analysis (n = 133). On occasions where both flower and concentrates were reported (n = 49), the average of the reported potencies for the two products was used.

#### 2.3.2. Craving

At each prompt, participants reported craving for alcohol as follows: “In the last 15 min: How strong was your urge to drink?” Participants could report from 1 (very slight or none) to 5 (very strong). To assess craving for cannabis, participants were asked, “In the last 15 min: How strong was your urge to use marijuana?” Participants could report from 1 (very slight or none) to 5 (very strong).

#### 2.3.3. Sleep

Once daily, in the morning report, participants were asked (1) “How many hours did you sleep last night?” Hours of sleep were reported using numeric entry. (2) “How would you rate the quality of your sleep?” Participants could respond with 0 (very bad), 1 (fairly bad), 2 (fairly good), 3 (very good).

### 2.4. Analytic procedures

#### 2.4.1. Primary analyses

Multilevel models (MLMs) were used to assess hypotheses. Primary outcomes of interest were sleep quality and sleep duration. The data were structured such that alcohol and cannabis use during the day predicted sleep quality and sleep duration that night (reported the following morning). Sleep quality was an ordinal variable and was evaluated with ordinal logistic regression using Proc GLIMMIX in SAS 9.4. Sleep duration was a continuous variable and was evaluated using Proc MIXED in SAS 9.4. Missing data was considered to be missing-at-random and models used restricted maximum likelihood (REML) to estimate model parameters using all available data. The data were comprised of assessment-level occasions nested within days and days nested within persons. However, occasions were aggregated to the day. Person-level random intercepts were specified. Person-level proportions of alcohol use and cannabis use days were also included, such that the use-day indicators reflect daily deviations from average use levels. This allows for separately assessing the relation between average levels of use (person-level effect) and daily deviations from average levels (day-level effect) on sleep quality and sleep duration.

The two primary outcomes (sleep quality and sleep duration) were modeled separately. For each, dichotomous cannabis use and alcohol use (reflecting any use of each substance that day) were entered as predictors, along with their interaction. The interaction allowed for examination of abstinent days, alcohol-only use, cannabis-only use, and alcohol-cannabis co-use. Notably, alcohol-cannabis co-use days included all days where both substances were reported. Thus, co-use in this study includes use on separate occasions within the same day and simultaneous use, where both substances are used at the same time or with overlapping effects. Participant age, sex at birth, weight at baseline, and PSQI score at baseline were included as covariates, along with day of week and day in study. Baseline PSQI score was included to adjust for possible systematic differences in outcomes (sleep, alcohol/cannabis use) due to schedule effects (e.g., increased sleep and alcohol/cannabis use on weekend days compared to weekdays). Day in study was included to adjust for potential systematic differences in outcomes over time (e.g., due to the effects of continued monitoring of behavior).

#### 2.4.2. Secondary analyses

A set of secondary analyses examined whether sleep quality and sleep duration (reported in the morning) predicted subsequent craving for alcohol and cannabis and engagement in alcohol and/or cannabis use that day. Sleep quality and sleep duration were included in separate models as predictors along with person-centered variables and covariates (age, sex at birth, weight, PSQI, day of week, and day in study). Models with craving for alcohol use and craving for cannabis use as outcomes used Proc MIXED in SAS 9.4. Craving outcomes were aggregated over the day. Engagement in day-level alcohol/cannabis use (vs. no use) was evaluated using binary logistic regression using Proc GLIMMIX in SAS 9.4. Sleep duration and sleep quality were person centered, meaning that person-averages were subtracted from day-level values to examine person-level and day-level effects separately.

## 3. Results

### 3.1. Descriptive

The average age of participants was 36.45 years (*SD*=10.99). Participants were 50 % female, majority White and non-Hispanic. Full information on participant demographics and frequency of alcohol/cannabis use days are presented in Table 1. Average scores on the AUDIT, CUDIT, and PSQI are presented in Table 1. On average, participants reported potentially hazardous levels of alcohol and cannabis use (AUDIT ≥ 8; CUDIT ≥ 13), as well as poor sleep quality (PSQI >5). Across the 60 days of EMA, participants completed 2456 morning reports (85.28%), 2624 afternoon reports (91.11%), 2612 evening reports (90.69%), and 2128 nighttime reports (73.89%). Overall participant compliance with the EMA protocol was 85.24%.

### 3.2. Impact of use on sleep quality and duration

Day-level alcohol use (b= −0.13, *p* = .304, 95 % CI: [−0.39, 0.12]), cannabis use (b= 0.36, *p*= .058, 95% CI: [−0.01, 0.73]), and their interaction, representing co-use, (b= −0.12, *p* = .615, 95 % CI: [−0.58, 0.34]) were not associated with sleep quality (see Table 2).

**Fig. 1.**
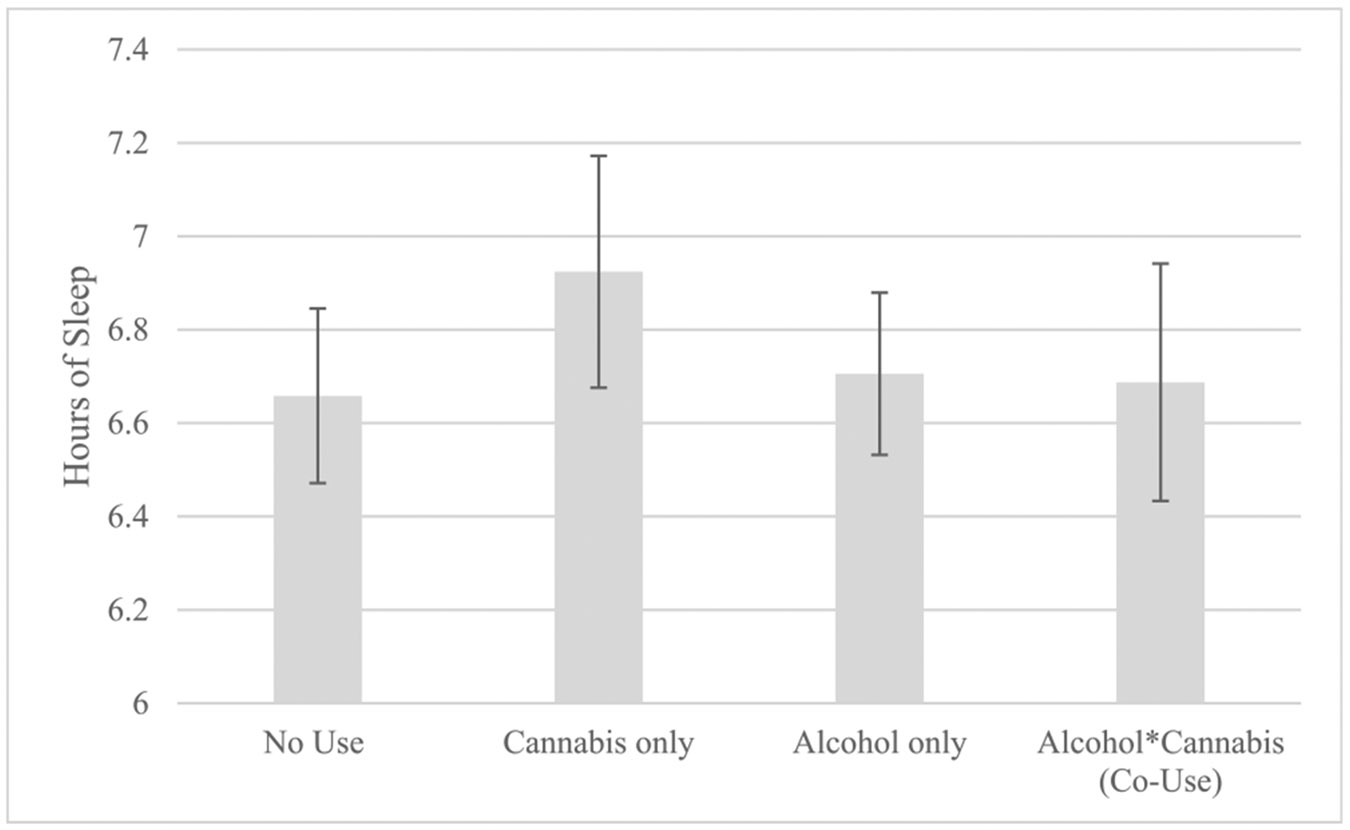
Model estimates for the multilevel model predicting self-reported sleep duration following nights of cannabis use, alcohol use, co-use of cannabis and alcohol, or no use of cannabis or alcohol. Error bars represent the standard error.

Day-level cannabis use was associated with longer sleep duration (b= 0.26, *p* = .006, 95 % CI: [0.08, 0.46]). Day-level alcohol use was not significantly associated with sleep duration (b=0.03, *p* = .647, 95 % CI: [−0.10, 0.17]). There was a significant interaction of day-level alcohol and cannabis use impacting sleep duration (b= −0.30, *p* = .016, 95 % CI: [−0.55, −0.06]) (see Table 3). Based on post-hoc comparisons, this interaction indicated that cannabis-only use days (*M*= 6.94, *SE*=0.25) were the only days associated with longer sleep duration (vs. alcohol-only days *M*=6.70, *SE*=0.17, comparison b= 0.27, *p* = .017; and nouse days *M*=6.67, *SE*=0.19, comparison b= 0.10 *p* = .009). Co-use days (*M*=6.67, *SE*=0.25) were no different from days with no use (b= −0.24, *p*= .171) (see Fig. 1).

Exploratory analyses examined whether the average THC potency consumed within a day was associated with sleep. Day-level THC potency was not associated with sleep quality (b=0.003, *p* = .601, 95 % CI: [−0.01, 0.02]), but was associated with longer sleep duration (b=0.02, *p* = .022, 95 % CI: [0.001,0.01]). Additional exploratory analyses examined whether the number of standard alcoholic drinks consumed within a day was associated with sleep, given the unexpected finding that day-level alcohol use was not associated with sleep quality or duration. Number of drinks consumed was not associated with sleep duration (b=−0.01, *p* = .592, 95 % CI [−0.04, 0.02]). A higher number of standard drinks was associated with worse sleep quality (b=−0.09. *p* = .012, 95 % CI [−0.16, −0.02]). However, when the interaction between day-level cannabis use and number of standard alcoholic drinks was added to the model to examine whether cannabis use moderated the effect of alcohol use on sleep quality, number of standard drinks became non-significant (b=−0.07, *p* = .112, 95 % CI [−0.15, 0.02]) and day-level cannabis use emerged as a significant predictor of sleep quality (b=0.40 *p* = .027, 95 % CI [0.04, 0.75]). Their interaction was not significant (b=−0.10, *p* = .158, 95 % CI [−0.24, 0.04]).

### 3.3. Sleep and next-day craving

Sleep quality (b=0.01, *p* = .350, 95 % CI: [−0.01, 0.04]) and duration (b= −0.001, *p* = .876, 95 % CI: [−0.01, 0.01]) were not associated with next-day craving for alcohol. Likewise, sleep quality (b= −0.007, *p*= .618, 95 % CI: [−0.03, 0.02]) and duration (b=0.006, *p*= .431, 95 % CI: [−0.01, 0.02]) were not associated with next-day craving for cannabis.

### 3.4. Sleep and next-day alcohol and cannabis use

Sleep quality was not associated with next-day engagement in alcohol/cannabis use (*OR*=1.03, 95 % CI: [0.86 – 1.23]). Longer sleep duration was associated with higher likelihood of engagement in alcohol use, cannabis use, or alcohol-cannabis co-use (vs. no use) (*OR*=1.13, 95 % CI: [1.02 – 1.26]) (see Table 4).

In exploratory models examining next-day alcohol/cannabis use outcomes separately, sleep quality was not associated with engagement in alcohol-only use (*OR*=1.03, 95 % CI: [0.86 – 1.23]), cannabis-only use (*OR*=1.15, 95 % CI: [0.89 – 1.48]), or alcohol-cannabis co-use (*OR*=1.02, 95 % CI: [0.77 – 1.35]). Sleep duration was associated with higher odds of cannabis-only use (*OR*=1.24, 95 % CI: [1.08 – 1.43]), but was not associated with engagement in alcohol-only use (*OR*=1.02, 95 % CI: [0.92 – 1.14]) or alcohol-cannabis co-use (*OR*=1.06, 95 % CI: [0.90 – 1.25]).

## 4. Discussion

This pre-registered study examined daily sleep quality and sleep duration among community adults engaged in regular alcohol and/or cannabis use. Significant proportions of participants in this sample had potentially hazardous levels of alcohol use and cannabis use at baseline. Despite not being recruited for sleep difficulties, a majority (67 %) of participants also reported significant sleep problems. Given the high prevalence of sleep problems among adults with regular alcohol and cannabis use, it is important to understand how alcohol-cannabis co-use influences sleep and how this is related to future alcohol and cannabis use patterns.

Primary analyses examined whether alcohol/cannabis use and co-use were associated with sleep quality and sleep duration. Particularly when cannabis was used without alcohol, cannabis use was associated with sleeping ~15 min longer, but not sleeping better (except in an exploratory model examining number of alcohol drinks consumed). In exploratory models examining THC potency, results were consistent with findings for day-level engagement in cannabis use, such that THC potency was associated with longer sleep duration but not sleep quality. These findings align with prior research among college students with moderate cannabis use indicating that cannabis use is associated with longer sleep duration (Sznitman et al., 2023). However, it differs from a study of community adults who used cannabis almost daily, which found no direct effect for cannabis use on sleep quality or duration (Wycoff et al., 2024). We speculate that the difference in findings relate to the sample. Specifically, cannabis may be linked to small, short-term improvements in sleep among those who use on occasion, but may not have the same associations with improved sleep among those who use near-daily. Notably, 15 min per night has limited clinical significance if these effects are not sustained over time. Findings may also differ for those with and without sleep complaints, as most participants in this and the Sznitman (2023) study reported problematic sleep, while participants in Wycoff et al. (2024)’s study reported, on average, “good” sleep quality. In either case, these findings underscore that it is important to assess for concurrent alcohol use when evaluating the impact of cannabis on sleep, as different associations with sleep duration were observed when alcohol-cannabis co-use days were separated from cannabis-only days.

Notably, in this study, we did not find that co-use attenuated alcohol’s negative impact on sleep, and we did not find an association between day-level alcohol use and sleep quality or duration. Although all prior studies examining co-use found that alcohol-only use was associated with at least one sleep parameter, findings differed around how alcohol impacted sleep. For example, Sznitman (2023) reported that, despite increased sleep discontinuity after alcohol use, sleep duration was longer after alcohol-only use. Wycoff (2024) reported lower sleep quality after alcohol-only use, but no impact on duration. Differences may be explained by alcohol consumption levels, as Graupensperger et al. (2021) and Wycoff et al. (2024) reported that a greater number of drinks was associated with worse sleep quality. Consistent with this, we observed an effect for alcohol consumption levels on sleep quality in an exploratory model, though this association was no longer significant once cannabis use was added to the model, suggesting that it was not a robust effect. The impacts of substance use on sleep are complex and likely impacted by which substances are used and the amount of use.

To assess potential bi-directional relationships, secondary analyses examined whether sleep quality or duration (reported in the morning) were associated with next-day craving for and use of alcohol/cannabis. We found that sleeping longer was associated with greater likelihood of next-day engagement in cannabis use, even when adjusting for day of the week, person-level proportions of alcohol use and cannabis use days, and other covariates. These findings align with prior work linking longer sleep duration to increased odds of cannabis use and prior findings that sleep duration is not associated with craving for cannabis (Wycoff et al., 2024). It is possible that longer sleep duration led to changes in behavior, for example staying up later the following night and participating in social activities, which could contribute to being more likely to engage in cannabis use despite not reporting higher craving. Alternatively, though speculative, sleeping longer following cannabis use may reinforce beliefs about using cannabis to aid with sleep, which could in turn lead to higher likelihood of engaging in subsequent use, potentially with sleep-related motives. This may be especially likely when individuals perceive substance-related gains in some areas of sleep following cannabis use (longer duration) yet are still experiencing difficulties in other domains and are seeking additional relief from sleep problems.

Limitations of this study include data collection occurring during the COVID-19 pandemic, which may have influenced participants’ use of alcohol or cannabis, or participants’ sleep. The number of participants was also relatively small, albeit adequate for the micro-longitudinal analyses proposed, especially given the extended EMA period. Analyses were temporally ordered, but all results are correlational. Participants were required to self-report alcohol/cannabis use via EMA and, to receive additional assessments at night, participants needed to report use or a plan to use; thus, we may have missed spontaneous use. It is also possible that some participants stopped or paused using alcohol or cannabis during the study after heavy use. Sleep disturbance is a common withdrawal symptom for both substances, so such periods of abstinence may have been associated with worse sleep quality or duration. This could possibly have obfuscated typical sleep patterns on days with no alcohol or cannabis use. Participants self-reported the THC concentration in the flower/dry herb or concentrate products they used. There is potential for inaccurate reporting of product potency, and this study did not collect information on other cannabinoids, such as CBD, which may also influence sleep. Additionally, this study collected limited self-report measures of sleep quality and duration. Sleep quality and duration were each measured once-daily using single items and this study did not include measures of sleep onset latency, number/length of nighttime awakenings, or next day fatigue.

Future self-report research may benefit from collecting information on the timing of cannabis and alcohol use in relation to daily sleep and wake times to understand whether longer duration of sleep following cannabis use is explained by falling asleep more quickly or staying asleep longer. Additionally, future studies should explore whether sleep patterns differ when alcohol and cannabis are used separately within the same day compared to when they are used with overlapping effects. As reported in Carpenter et al. (2024), on most of the co-use days in this sample, participants reported alcohol and cannabis use at the same prompt, such that effects would likely overlap. While self-reported sleep indices are clinically meaningful, future work should also examine the relationship between alcohol-cannabis co-use and physiological indicators of sleep patterns, for example those collected through actigraphy or polysomnography. There is also potential for passive methods of data collection (e.g., transdermal alcohol concentration, actigraphy) to reduce participant burden and provide additional information that may be complementary with self-report. However, it is important to note that such methods have their own limitations.

Community adults who are relatively sleep restricted and use alcohol/cannabis on a weekly basis report sleeping ~15 min longer on days of cannabis (but not alcohol or combined alcohol/cannabis) use. Unfortunately, experimental studies (Gorelick et al., 2013) and naturalistic research among those who use cannabis almost daily (Wycoff et al., 2024) indicate that this small, potential short-term benefit of cannabis use on sleep is unlikely to persist over time. This perceived potential benefit is also concerning because it may perpetuate both cannabis use and sleep problems. Longitudinal and experimental studies teasing apart how and for whom cannabis impacts sleep (either negatively or positively) are strongly encouraged. In particular, we recommend testing frequency of cannabis use and diagnostic criteria for insomnia as moderators of day-level associations between sleep and cannabis use.

## Supplementary Material

1

## Figures and Tables

**Table 1:** Participants’ demographics and clinical characteristics (*N* = 48).

	n	%
Sex		
Male	24	50%
Female	24	50%
Ethnicity		
Hispanic	9	18.8 %
Race		
White	39	81.25 %
Black	3	6.25 %
More than one race	2	4.17 %
Another race	1	2.08 %
Did not disclose race	3	6.25 %
Alcohol/cannabis use during EMA		
No use reported	0	0%
Reported cannabis-only use days	6	12.5 %
Reported alcohol-only use days	17	35.42 %
Reported co-use days	25	52.08 %
Day-level use during EMA		
No use day	1025	36.87 %
Cannabis-only day	694	24.96 %
Alcohol-only day	744	26.76 %
Co-use day	317	11.40 %
Sleep duration during EMA		
<7 h	942	38.70 %
	*M* or *n*	*SD* or *%*
AUDIT total score	9.50	9.23
AUDIT ≥ 8	20	41.67 %
CUDIT total score	10.86	7.49
CUDIT ≥ 13	24	31.43 %
PSQI total score	8.62	4.52
PSQI > 5	32	66.66 %

*Note*: The sample consisted of 48 participants who completed 60 days of ecological momentary assessment (EMA). Alcohol Use Disorder Identification Test (AUDIT), Cannabis Use Disorder Identification Test (CUDIT), Pittsburgh Sleep Quality Index (PSQI). CUDIT total scores were only available for 35 participants due to missing data.

**Table 2:** Use predicting sleep quality.

	b	95 % CI	*p*	*SE*
Intercept: Sleep Quality = 3 (very good)	−2.41	−5.44, 0.63	.117	1.50
Intercept: Sleep Quality = 2 (fairly good)	1.31	−1.72, 4.34	.387	1.50
Intercept: Sleep Quality = 1 (fairly bad)	3.81	0.77, 6.85	.015	1.50
Day-level alcohol use	−0.13	−0.39, 0.12	.304	0.13
Day-level cannabis use	0.36	−0.01, 0.73	.058	0.19
Day-level alcohol*cannabis use (co-use)	−0.12	−0.58, 0.34	.615	0.24
Person-level proportion of alcohol use days	−0.99	−3.01, 1.04	.338	1.03
Person-level proportion of cannabis use days	−0.91	−2.40, 0.58	.232	0.76
Age	0.02	−0.02, 0.06	.351	0.02
Sex	0.30	−0.64, 1.24	.529	0.48
Weight	0.002	−0.01, 0.01	.711	0.006
**PSQI**	**−0.16**	**−0.27, −0.05**	**.003**	**0.05**
**Study Day**	**0.01**	**0.001, 0.01**	**.025**	**0.003**
Weekday: Friday	−0.01	−0.33, 0.31	.953	0.16
Weekday: Monday	−0.15	−0.46, 0.16	.358	0.16
Weekday: Saturday	−0.07	−0.39, 0.25	.677	0.16
Weekday: Thursday	−0.25	−0.57, 0.06	.116	0.16
Weekday: Tuesday	−0.24	−0.56, 0.08	.137	0.16
Weekday: Wednesday	−0.31	−0.62, 0.005	.054	0.16

*Note*: Very bad (0) is the reference category for sleep quality. Sunday is the reference category for weekday. Rows in bold are significant at *p <* .05.

**Table 3:** Use predicting sleep duration.

	b	95 % CI	*p*	*SE*
Intercept	6.12	3.47, 8.76	< .0001	1.31
Day-level alcohol use	0.03	−0.10, 0.17	.647	0.06
**Day-level cannabis use**	**0.27**	**0.08, 0.46**	**.006**	**0.10**
Day-level alcohol*cannabis use (co-use)	**−0.30**	**−0.55, −0.06**	**.016**	**0.13**
Person-level proportion of alcohol use days	0.62	−1.19, 2.42	.493	0.89
Person-level proportion of cannabis use days	−1.01	−2.31, 0.30	.126	0.65
Age	0.01	−0.02, 0.05	.466	0.02
Sex	0.40	−0.45, 1.24	.347	0.42
Weight	0.001	−0.01, 0.01	.877	0.01
PSQI	−0.07	−0.16, 0.03	.146	0.05
**Study Day**	**0.003**	**0.0001, 0.01**	**.042**	**0.001**
Weekday: Friday	−0.07	−0.23, 0.10	.409	0.08
Weekday: Monday	−0.01	−0.17, 0.16	.917	0.08
Weekday: Saturday	−0.04	−0.20, 0.13	.667	0.08
**Weekday: Thursday**	**−0.21**	**−0.37, −0.04**	**.013**	**0.08**
Weekday: Tuesday	−0.12	−0.28, 0.05	.158	0.08
Weekday: Wednesday	−0.14	−0.31, 0.02	.085	0.08

*Note*: Sunday is the reference category for Weekday. Rows in bold are significant at *p <* .05.

**Table 4:** Sleep duration predicting next-day use (0 = neither alcohol nor cannabis, 1 =either/both).

	OR	95 % CI	*p*	*SE*
Intercept	8.03	0.02, 2825.1	.477	2.91
**Sleep hours**	**1.13**	**1.02, 1.26**	**.018**	**0.05**
Person-centered sleep hours	0.75	0.43, 1.31	.313	0.29
Age	1.02	0.96, 1.10	.481	0.03
Sex	0.65	0.15, 2.83	.562	0.75
Weight	1.003	0.98, 1.02	.763	0.01
PSQI	1.01	0.86, 1.20	.887	0.09
**Study Day**	**0.98**	**0.97, 0.99**	**< .0001**	**0.003**
Weekday: Friday	1.36	0.91, 2.05	.139	0.21
Weekday: Monday	0.74	0.49, 1.12	.152	0.21
Weekday: Saturday	1.26	0.83, 1.91	.271	0.21
Weekday: Thursday	0.96	0.63, 1.44	.832	0.21
Weekday: Tuesday	0.83	0.55, 1.25	.366	0.21
Weekday: Wednesday	0.80	0.53, 1.21	.294	0.21

*Note*: Sunday is the reference category for weekday. Rows in bold are significant at *p <* .05.

## Appendix A. Supporting information

Supplementary data associated with this article can be found in the online version at doi:10.1016/j.drugalcdep.2025.112946.
