# Supplementary material for: Sleep quality and duration following the use and co-use of alcohol and cannabis in the daily life of community adults
Source: Drug Alcohol Depend. Author manuscript; Available in PMC 2026 Jan 30. (PMC12857193; doi:10.1016/j.drugalcdep.2025.112946)
Supplement: 1 [file NIHMS2130936-supplement-1.docx]

|  |  | |  | |  | | |  | |  | |  | |  | |  |
| --- | --- | --- | --- | --- | --- | --- | --- | --- | --- | --- | --- | --- | --- | --- | --- | --- |
|  | | Reported cannabis-only use days (n=6) | | | | Reported alcohol-only use days (n=17) | | | Reported co-use days (n=25) | | | | Total sample (n=48) | | | |
|  | | *n* | | *%* | | *n* | *%* | | *n* | | *%* | | *n* | | *%* | |
| Sex | |  | |  | |  |  | |  | |  | |  | |  | |
| Male | | 3 | | 50% | | 7 | 41.18% | | 14 | | 56% | | 24 | | 50% | |
| Female | | 3 | | 50% | | 10 | 58.82% | | 11 | | 44% | | 24 | | 50% | |
| Ethnicity | |  | |  | |  |  | |  | |  | |  | |  | |
| Hispanic | | 1 | | 16.67% | | 2 | 11.75% | | 6 | | 24% | | 9 | | 18.8% | |
| Race | |  | |  | |  |  | |  | |  | |  | |  | |
| White | | 5 | | 83.33% | | 14 | 82.35% | | 20 | | 80% | | 39 | | 81.25% | |
| Black | | 0 | | 0% | | 2 | 11.77% | | 1 | | 4% | | 3 | | 6.25% | |
| More than one race | | 0 | | 0% | | 0 | 0% | | 2 | | 8% | | 2 | | 4.17% | |
| Another race | | 1 | | 16.67% | | 0 | 0% | | 0 | | 0% | | 1 | | 2.08% | |
| Did not disclose race | | 0 | | 0% | | 1 | 5.88% | | 2 | | 8% | | 3 | | 6.25% | |
| Day-level use during EMA | |  | |  | |  |  | |  | |  | |  | |  | |
| No use day | | 101 | | 28.06% | | 494 | 49.85% | | 430 | | 30.09% | | 1,025 | | 36.87% | |
| Cannabis-only day | | 259 | | 71.94% | | 0 | 0% | | 435 | | 30.44% | | 694 | | 24.96% | |
| Alcohol-only day | | 0 | | 0% | | 497 | 50.15% | | 247 | | 17.28% | | 744 | | 26.76% | |
| Co-use day | | 0 | | 0% | | 0 | 0% | | 317 | | 22.18% | | 317 | | 11.40% | |
| Sleep duration during EMA | |  | |  | |  |  | |  | |  | |  | |  | |
| < 7 hours | | 127 | | 40.45% | | 246 | 26.39% | | 569 | | 47.90% | | 942 | | 38.70% | |
|  | | *M* or *n* | | *SD* or *%* | | *M* or *n* | *SD* or *%* | | *M* or *n* | | *SD* or *%* | | *M* or *n* | | *SD* or *%* | |
| AUDIT total score | | 3.17 | | 7.28 | | 7.71 | 6.50 | | 12.24 | | 10.36 | | 9.50 | | 9.23 | |
| AUDIT ≥ 8 | | 1 | | 16.67% | | 6 | 35.29% | | 13 | | 52% | | 20 | | 41.67% | |
| CUDIT total score | | 15.5 | | 9.83 | | 2.00 | 0.82 | | 11.16 | | 6.38 | | 10.86 | | 7.49 | |
| CUDIT ≥ 13 | | 3 | | 50% | | 0 | 0% | | 8 | | 32% | | 24 | | 31.43% | |
| PSQI total score | | 8.50 | | 5.17 | | 7.24 | 4.07 | | 9.60 | | 4.59 | | 8.62 | | 4.52 | |
| PSQI >5 | | 4 | | 66.67% | | 10 | 58.82% | | 18 | | 72% | | 32 | | 66.66% | |

*Note*: The sample consisted of 48 participants who completed 60 days of ecological momentary assessment (EMA). Alcohol Use Disorder Identification Test (AUDIT), Cannabis Use Disorder Identification Test (CUDIT), Pittsburgh Sleep Quality Index (PSQI). CUDIT total scores were only available for 35 participants due to missing data.
